# Acitretin-Induced Repigmentation of Gray Hair: A Case Report

**DOI:** 10.7759/cureus.58261

**Published:** 2024-04-14

**Authors:** Eunice Y Chow, Thomas G Salopek

**Affiliations:** 1 Medicine/Dermatology, University of Alberta, Edmonton, CAN

**Keywords:** hair graying, canities, hair repigmentation, retinoids, acitretin

## Abstract

Acitretin is an oral retinoid with alopecia as a possible adverse effect. However, repigmentation of the hair color after acitretin is not a well-documented phenomenon. Herein, we introduce a case where a patient’s hair color darkened after a course of acitretin.

## Introduction

In Canada, acitretin is indicated for the treatment of severe psoriasis and other disorders of keratinization (this category of disease includes pityriasis rubra pilaris (PRP)) [1], while in the USA, the Food and Drug Administration has only approved its use for severe psoriasis [2]. Among its listed adverse effects within the category of “skin and subcutaneous tissue disorders” is alopecia. The mechanism of graying of the hair is complex, and there are no medical treatments for repigmentation of hair. Through a literature search, there have only been two other documented cases of hair repigmentation as a result of acitretin [3,4]. Here, we report a third case of hair repigmentation secondary to acitretin.

## Case presentation

An 80-year-old man presented to the University Dermatology Clinic with a several-month history of a widespread eruption, palmoplantar keratoderma, and hypertrophic nail changes. He was ultimately diagnosed with PRP. His medical history included hypertension and diabetes. His medication list at the time included aspirin, betamethasone valerate 0.1% cream, hydrocortisone valerate 0.2% cream, indapamide, lisinopril, and metformin. To manage his PRP, shortly after he was diagnosed, he was initially put on 40 mg daily of acitretin (~0.5 mg/kg/day) along with betamethasone valerate 0.1% ointment for six months. Due to an incomplete response, the dose was increased to 50 mg daily (~0.6 mg/kg/day). Within two months at the higher dosage, the widespread eruption on his trunk had cleared, and the thick keratoderma of his palms and soles dramatically decreased, such that only the finger and toenail dystrophy and a few red patches on the face and neck persisted. Ultimately, after approximately 18 months of therapy, he had complete resolution of all cutaneous and nail changes. By around the 20th month, he started noticing that his hair, which had been completely white for many decades, had started to darken compared to his previous hair color. He did not have any other adverse effects from the acitretin.

**Figure 1:**
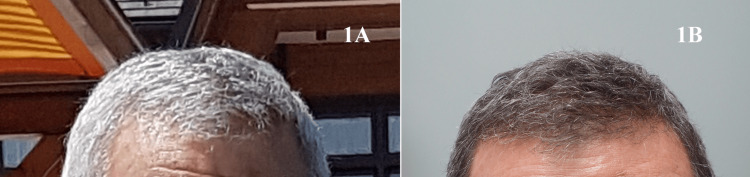
(1A) White/light gray hair before acitretin therapy; (1B) repigmented dark gray hair after a course of acitretin

On examination, the darkened gray hair was throughout his scalp. There were no notable changes in the hair texture or hair loss. This was not concerning to the patient and he continued with his therapy (Figure 1).

In the end, he gradually tapered off acitretin over several months, so he had a total of 24 months of acitretin. He was lost to follow-up. As such, it is unknown as to whether the repigmentation of his hair persisted upon discontinuation of the drug.

## Discussion

Hair graying, also known as canities, had been thought of as a natural irreversible part of aging due to the absence or decrease in melanin produced by melanocytes within the anagen hair bulb or a decrease in the deposition of melanin within the hair shafts [5]. There is a strong genetic component to hair graying due to at least 68 human genes described that are related to the hypopigmentation and depigmentation of hair [5]. In addition to genes influencing hair graying, there are also a number of other environmental factors that act on the hair follicle, such as pollution, UV radiation, psycho-emotional factors, nutrition, metabolism, exposure to cigarette smoke, racial/ethnic, gender, and inflammation, to cause graying of the hair [6]. A number of pathways are involved in the regulation of melanogenesis and any factor that may affect these pathways may influence the graying or repigmentation. These signaling pathways include the Wnt/ꞵ-catenin, MC1R (melanocortin 1 receptor), SCF/C-KIT (stem cell factor/tyrosine kinase receptor), EDN/EDNRB (endothelin/endothelin receptor B), PI3K/AKT (phosphoinositide 3-kinase/ serine/threonine-specific protein kinase), TGF-ꞵ (transforming growth factor ꞵ), and MITF (melanocyte-inducing transcription factor) [6]. Other mechanisms that may influence the regulation of hair pigmentation include the sympathetic/sensory nerves, certain neurotransmitters (e.g., calcitonin gene-related peptide (CGRP), substance P (SP), and vasoactive intestinal peptide (VIP)) and the interaction between adipose tissue and the hair follicle [7].

A multitude of medications have been reported to induce gray hair repigmentation (Table 1) [7,8]. One proposed mechanism for acitretin-related hair repigmentation is through acitretin’s action on lowering IL-6, hence potentially causing hair repigmentation through an anti-inflammatory action [8]. A review by Feng et al. [7] further explored the possible mechanism behind hair repigmentation from systemic retinoids and suggested that it is through acitretin’s impact on retinoic acid metabolism, potentially increasing the level of retinoic acid (which in turn upregulates the C-KIT receptor and sensitizes melanocyte stem cells to the KIT-ligand) that changes the hair color. However, considering that there are many patients who take acitretin and very few reports of hair repigmentation after acitretin, there may be another unknown factor that predisposes patients to this effect. There is one case report of etretinate-induced hair repigmentation [9].

**Table 1:** List of agents reported to induce hair repigmentation Sources: [7,8]

Category of medications	List of agents reported to induce hair repigmentation
Monoclonal antibody drugs	Anti-PD-1/PD-L1 therapy, dupilumab, adalimumab, secukinumab, ustekinumab, brentuximab
Tyrosine kinase inhibitors	Dasatinib, imatinib, sorafenib, erlotinib
Immunomodulatory drugs	Lenalidomide, thalidomide
Immunosuppressants	Cyclosporine, prednisone
Others	Retinoids, interferon-𝛂2+ribavirin+pegylated- interferon, 5-flurouracil+leucovorin+levamisole+cisplantinum, 8-methoxy psoralen, latanoprost, tamoxifen, levodopa, clofazimine, L-thyroxine, acetylcholinesterase inhibitor, L-DOPA, cerebrolysin, and multiple combinations of vitamin supplements

The two other case reports of acitretin-induced hair repigmentation included a 70-year-old woman given 25 mg acitretin daily for psoriasis [3] and a 61-year-old man given 25 mg acitretin daily for PRP [4]. In the former case, the patient noticed hair repigmentation after six months, and in the second case, the patient noticed repigmentation “over the course of one year.” Our patient noticed hair repigmentation after around 20 months of therapy on an average of 50 mg of acitretin daily. Our patient presented with hair repigmentation later than the other two reported cases, and our patient was also on a higher dosage of acitretin. Moreover, unlike the other two cases of acitretin-induced hair repigmentation [3,4], our patient did not notice any curling of his hair in addition to the increased pigmentation. Moreover, the onset was much later, while previous reports suggest repigmentation with several months to a year into treatment. Although one of the case reports also had PRP like our patient, this condition is not known to have hair changes. While alopecia is a known adverse effect of acitretin, neither our patient nor the other two cases reported hair loss [3,4].

Unfortunately, our patient was lost to follow-up with clearance of his primary dermatologic condition; as such, we do not know if the repigmentation of his hair was permanent or reverted to gray upon discontinuation of the acitretin. Similarly, the two prior cases of repigmentation did not comment on this.

## Conclusions

We report a patient with a darkening of his hair after acitretin therapy. Although there are plausible mechanisms of action for this effect, it is still unclear how acitretin causes the repigmentation of hair and who might be predisposed to this effect. This phenomenon, however, may lead future researchers to better understand aging and hair graying and its reversal.
